# SARS coronavirus protein 7a interacts with human Ap_4_A-hydrolase

**DOI:** 10.1186/1743-422X-7-31

**Published:** 2010-02-09

**Authors:** Natalia Vasilenko, Igor Moshynskyy, Alexander Zakhartchouk

**Affiliations:** 1 Vaccine and Infectious Disease Organization (VIDO), University of Saskatchewan, 120 Veterinary Road, Saskatoon, SK S7N 5E3, Canada

## Abstract

The SARS coronavirus (SARS-CoV) open reading frame 7a (ORF 7a) encodes a 122 amino acid accessory protein. It has no significant sequence homology with any other known proteins. The 7a protein is present in the virus particle and has been shown to interact with several host proteins; thereby implicating it as being involved in several pathogenic processes including apoptosis, inhibition of cellular protein synthesis, and activation of p38 mitogen activated protein kinase. In this study we present data demonstrating that the SARS-CoV 7a protein interacts with human Ap_4_A-hydrolase (asymmetrical diadenosine tetraphosphate hydrolase, EC 3.6.1.17). Ap_4_A-hydrolase is responsible for metabolizing the "allarmone" nucleotide Ap_4_A and therefore likely involved in regulation of cell proliferation, DNA replication, RNA processing, apoptosis and DNA repair. The interaction between 7a and Ap_4_A-hydrolase was identified using yeast two-hybrid screening. The interaction was confirmed by co-immunoprecipitation from cultured human cells transiently expressing V5-His tagged 7a and HA tagged Ap_4_A-hydrolase. Human tissue culture cells transiently expressing 7a and Ap_4_A-hydrolase tagged with EGFP and Ds-Red2 respectively show these proteins co-localize in the cytoplasm.

## Background

Severe acute respiratory syndrome coronavirus (SARS-CoV) has been shown to be the etiological agent for the global SARS outbreak in the winter 2002/2003 that affected about 30 countries [1].

SARS-CoV is an enveloped, positive-sense RNA virus with ~30 kb genome. It contains 14 potential ORFs. Some of these ORFs encode proteins that are homologues to the structural proteins founded in other coronaviruses, namely the replicase (ORF 1a and 1b), membrane, nucleocapsid, envelope and spike proteins [2,3]. Other ORFs encode group-specific or accessory proteins which are unique to SARS-CoV.

Accessory proteins are not necessary for viral replication in cell culture systems and in mice, but may be important for virus-host interactions and thus may contribute to viral strength and/or pathogenesis *in vivo *[4-6].

Protein 7a (also known as ORF 8, U122 and X4 protein [2,3,7]), 122 amino acids in length, shows no significant similarity to any other viral or non-viral proteins. The ORF 7a gene is conserved in all SARS-CoV strains [8], and sequence analysis predicts that ORF 7a encodes a type I transmembrane protein. The crystal structure of the luminal domain of the 7a protein has been resolved, revealing a structure unexpectedly similar in fold and topology to members of the immunoglobulin superfamily [9]. It has been demonstrated that 7a is incorporated into SARS-CoV particles by interacting with viral structural proteins E and M [10,11]. In addition, 7a interacts with the viral proteins 3a and S [10,12], and these proteins may form a complex during viral infection.

Recombinant mutant SARS-CoV lacking the 7a gene is completely viable in cultural cells and mice [4]; therefore, 7a protein is dispensable for virus growth and replication but may play role in virus-host interactions.

The 7a protein seems to have diverse biological functions in cultured cells. Over-expression of ORF 7a induces apoptosis via the caspase-dependent pathway [13] and inhibits cellular protein synthesis by activation of p38 MAPK [14]. The induction of apoptosis by the 7a protein is dependent on its interaction with the Bcl-X_L _protein and other pro-survival proteins (Bcl-2, Bcl-w, Mcl-1 and A1) [15]. In addition, 7a can block cell cycle progression at the G0/G1 phase via the cyclin D3/pRb pathway [16]. Also, interaction between 7a and hSGT (human small glutamine-rich tetricopeptide repeat containing protein) has been demonstrated although the biological significance of this interaction needs to be further elucidated [17]. Taken together, these observations suggest that the 7a protein interacts with several host cell proteins and may play a role in the SARS-CoV pathogenesis.

We performed a yeast-two-hybrid screening using a commercially prepared human lung cDNA library as the source of the "prey" cDNAs and using a full-length ORF 7a as the "bait". Among the potential novel 7a interacting partners, Ap_4_A-hydrolase was identified. Its interaction with 7a was confirmed by co-immunoprecipitation and co-localization experiments in transiently transfected cultured human cells.

Ap_4_A-hydrolase belongs to the Nudix (nucleoside diphosphate linked to x) hydrolases, which are a superfamily of enzymes required for maintenance of physiological homeostasis by metabolizing signaling molecules and potentially toxic substances. Ap_4_A-hydrolase is found in all higher eukaryotes and contributes to regulation of the intracellular level of "allarmone" nucleotide Ap_4_A [18,19]. It is an asymmetrically-cleaving enzyme, catalyzing the reaction (Ap_4_→ATP+AMP). The intracellular concentration of Ap_4_A has been shown to increase in cells after heat, oxidative, nutritional or DNA damage stresses [20]. A recent study demonstrated that Ap_4_A-hydrolase belongs to the transcriptional regulation network in immunologically activated mast cells and that it is involved in regulation of the MITF gene in cardiac cells [21].

## Results

### Identification of human cellular proteins interacting with SARS-CoV protein 7a

In order to identify cellular proteins that interact with the 7a protein, we performed a yeast two-hybrid screening of the Hybrid Hunter™ Premade cDNA library (human adult lung) constructed in pYESTrp2 (Invitrogen). Approximately 1 × 10^6 ^transformants were screened. From these, 35 clones were able to grow in the selective media (-Trp, -Leu, -His) and gave a strong signal when analyzed for β-galactosidase activity. Plasmid DNA from the positive yeast clones was purified and used for transformation of the *E. coli *strain DH5α. Next, the DNA of the plasmids was sequenced and analyzed using BLAST. One of the clones contained a complete ORF that showed sequence identity to the human Ap_4_A-hydrolase gene [GenBank: U30313].

### Co-immunoprecipitation

**Figure 1:**
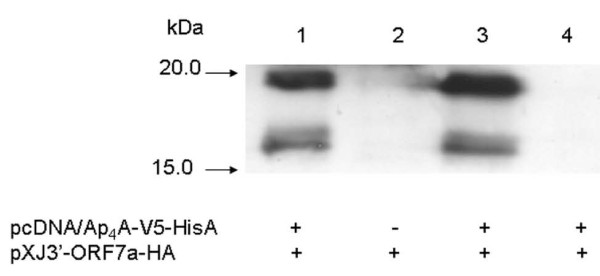
**Co-immunoprecipitation of Ap_4 _A-hydrolase and the SARS-CoV 7a protein**. HEK 293 cells were transiently transfected with pcDNA3.1/V5-His-Ap_4_A and/or pXJ-7a-HA. Proteins from cell lysates were immunoprecipitated with monoclonal anti-V5 antibodies (lanes 1 and 2) or unrelated monoclonal anti-β-actin antibody (lane 4). After immunoprecipitation, samples were subjected to 12% SDS-PAGE, transferred to nitrocellulose membrane and probed with monoclonal anti-HA antibodies. Lane 3 represents Western blotting of cell lysates without immunoprecipitation as a control.

To verify interactions established by the yeast two-hybrid screening, we used a co-immunoprecipitation assay. HEK 293 cells were either transfected with pXJ3'-ORF7a-HA, or co-transfected with both pXJ3'-ORF7a-HA and pcDNA3.1/V5-His -Ap_4_A. The transfected cells were lysed 24-48 h after transfection. Protein extracts were immunoprecipitated using monoclonal Anti-V5 antibody (Fig. 1, lanes 1 and 2). As a negative control, the same protein extracts were immunoprecipitated using an unrelated antibody - mouse monoclonal anti-β-actin (Fig. 1, lane 4). Antibody-antigen complexes were immobilized on protein A and G-sepharose bead mixture. The immobilized proteins were eluted by boiling the beads in SDS-PAGE loading buffer. The samples were subsequently analyzed by Western blot, using an anti-HA monoclonal antibody, to detect HA tagged co-immunoprecipitated proteins. As shown in Fig. 1, the HA-tagged 7a protein co-immunoprecipitated with V5-tagged Ap_4_A-hydrolase. As described earlier [12], the 7a protein was detected in the transfected cells in two forms: unprocessed and processed. The 7a protein was not detected when unrelated antibody was used for co-immunoprecipitation. Our results indicate that 7a specifically interacts with Ap_4_A-hydrolase in human cells.

### Subcellular co-localization of 7a and Ap_4_A-hydrolase

There is no consensus opinion in subcellular localization of 7a protein [for review see [11]]. 7a protein was found co-locolaze with trans-Golgi marker, Golgin97 [9], in the Golgi compartment [14]. Other reports demonstrated co-localization with endoplasmic reticulum marker GRP94 or the intermediate compartment marker Sec 31 [7,17]. Since the co-immunoprecipitation experiment showed interaction between HA- tagged 7a and V5-tagged Ap_4_A-hydrolase, we hypothesized that these proteins may co-localize in human cells. To visualize these proteins, we tagged them with fluorescent proteins: 7a with EGFP and Ap_4_A-hydrolase with DsRed2. HEK 293 cells were co-transfected with pEGFP-C2-ORF7a and pDsRed2-Ap_4_A, and sub-cellular distribution of proteins was examined by confocal microscopy.

**Figure 2:**
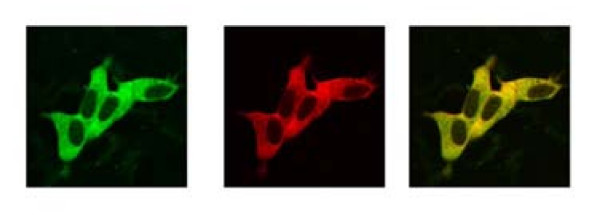
**Co-localization of EGFP-tagged 7a and DsRed2-tagged Ap_4 _A-hydrolase in transiently transfected HEK 293 cells**. The cells were grown on 35 mm glass bottom culture dishes and were subjected to confocal microscopy using a Zeiss LSM410 microscope, as described in *Material and Methods*. Both signals were detected simultaneously; separate images were taken and superimposed. Left panel shows the cellular distribution of transiently expressed EGFP-tagged 7a. Middle panel shows red fluorescence of DsRed2-tagged Ap_4_A-hydrolase in the same cells. Right panel represents a superimposition of both images.

Our data demonstrated that both proteins had similar cytoplasmic localization (Fig. 2, left and middle panels). Co-localization analysis showed an overlap in the subcellular distribution of both proteins, as can be seen by the appearance of the yellow color in the overlaid image (Fig. 2, right panel).

## Discussion

The molecular mechanisms of SARS-CoV pathogenesis are not fully understood and the contribution of the group-specific proteins, also known as accessory proteins, to this process has not been completely determined. The study of interactions between viral and cellular proteins may help to elucidate molecular mechanisms of SARS-CoV pathogenicity. In our study, we have shown an interaction between the 7a protein and Ap_4_A-hydrolase, an enzyme involved in a number of biological processes [18-21].

Interaction between 7a protein and Ap_4_A-hydrolase was initially found in the yeast two-hybrid system and then confirmed in human cells. Human lung cDNA library was used for initial screening because although SARS-CoV may affect various organs and tissues, its primary site of infection is the respiratory tract [22,23]. The interaction that has been found in yeast was confirmed by co-immunoprecipitation analysis carried out with the recombinant proteins extracted from the transfected HEK 293 cells (Fig. 1) and confocal microscopy of these cells (Fig. 2).

The substrate of Ap_4_A-hydrolase, Ap_4_A, can be found in all organisms including Archae and humans, and it is a side product of protein synthesis catalyzed by amino-acyl-tRNA synthetases [21]. Although the biological significance of Ap_4_A is not fully understood, it has been proposed to be an intracellular and extracellular signaling molecule affecting cell proliferation and DNA replication [18,24,25], RNA processing [26,27], heat shock and oxidative stress [19,28], apoptosis and DNA repair [29]. As an extracellular component, Ap_4_A may play an important role as a neurotransmitter in the cardiovascular system [30,31]. In bacteria, Ap_4_A levels may also play a role in invasion [32,33].

Multiple pathways may be involved in the regulation of apoptosis and cell cycle arrest during SARS-CoV infection. It is well established that over-expression of 7a protein in cultured cells induces apoptosis via increasing level of caspase-3 protease activity [13]. Interestingly, Ap_4_A is also involved in caspase activation [34]. Elevation of Ap_4_A level could be achieved by direct up-regulation of the amino-acyl-tRNA synthetases, or down-regulation of the Ap_4_A-hydrolases, or both, in response to the appropriate signal. Our data indicate that 7a interacts with Ap_4_A-hydrolase and thus may down-regulate the Ap_4_A-hydrolase enzymatic activity. Theoretically, this would result in an increase of Ap_4_A level and contribute to the induction of apoptosis. Further study is required to confirm this hypothesis.

Protein 7a has been reported to interact with Bcl-2 protein and several other prosurvival members of Bcl-2 family [16]. Anti-apoptotic Bcl-2 protein inhibits caspase-dependent apoptosis induced by SARS-CoV infection but does not affect viral replication kinetics [35]. It has also been shown that Ap_4_A-induced apoptosis is accompanied by a significant reduction in the level of anti-apoptotic Bcl-2 protein in mammalian cells [36]. Since Bcl-2 is a substrate of caspase-3 in myeloid leukemic cells, Vartanian and co-workers suggested that Ap_4_A is involved in the cascade of events leading to caspase activation [34,36].

Cell cycle disregulation is a common response of host cells to many viral infections. Some SARS-CoV proteins, including nucleocapsid N [37], 3b [38] and 7a [15] block cell cycle progression at the G0/G1 phase. As for 7a, it has been demonstrated that its expression inhibits phosphorylation of one of the key regulators of cell cycle progression - Rb protein [15]. The hyperphosphorylation of Rb allows activation of E2F family of transcription factors, permitting the transcription of the S phase genes [39]. The inhibition of Rb phosphorylation by 7a suggests that the expression of G1 cyclin/cdk complexes is suppressed. On the other hand, flow cytometric analysis indicated an involvement of Ap_4_A at an early stage of G1/S arrest by dephosphorylation of pRb via reduction of CDK 2 activity [36].

Taken together, these data suggest that 7a and Ap_4_A-hydrolase may be involved in a common cascade of mechanisms leading to cell cycle arrest and apoptosis. Further insight into the functions of both 7a protein and Ap_4_A-hydrolase will still have to be gained before the physiological importance of their interactions can be elucidated. In addition, more experiments are required to determine the significance of the obtained data. For instance, co-immunoprecipitation and co-localization assays need to be repeated using SARS-CoV infected cells and protein-specific anti-sera. Also, it will be useful to infect cells with SARS-CoV carrying a deletion of the 7a gene [4] in order to study apoptosis and cell cycle regulation in the infected cells.

## Conclusions

The study of interactions between viral and cellular proteins may help to elucidate molecular mechanisms of SARS-CoV pathogenicity. In the present paper, we have shown an interaction between the SARS-CoV 7a protein and Ap_4_A-hydrolase, an enzyme involved in a number of biological processes. These two proteins may participate in common pathways leading to cell cycle arrest and apoptosis. The biological significance of the interaction between 7a and Ap_4_A-hydrolase needs to be elucidated.

## Materials and methods

### DNA constructs

The plasmid pXJ3'-ORF7a-HA containing full-length SARS-CoV ORF 7a with C-terminal HA-tag was a generous gift from Dr. Yee-Joo Tan (Institute of Molecular and Cell Biology, Singapore) and has been described elsewhere in detail [12].

In our study, the ORF 7a gene was amplified from RNA of SARS-CoV-infected Vero E6 cells (strain Tor 2) using a one step RT PCR kit (Qiagen, Mississauga, Canada) (primers 5'-GGGGTACCATGAAAATTATTCTCTTCCT-3' and 5'-GGAATTCTCATTCTGTCTTTCTCTTAA-3') and cloned into the *Kpn *I and *Eco*R I sites (underlined) of the pH5L vector [40] to create pH5L-ORF7a. Next, ORF 7a was amplified from the plasmid pH5L-ORF7a using primers 5'-CGGAATTCATGTTTCATCTTGTTGACTT-3' and 5'-CGCTCGAGTTATGGATAATCTAACTCCA-3'. The PCR-product was digested with *Eco*R I and *Xho *I (recognition sites are underlined) and subcloned into pHybLex/Zeo (Invitrogen, Burlington, Canada) in frame with LexA to create the "bait" pHybLex/Zeo-ORF7a plasmid for yeast two-hybrid library screening. Then, pH5L-ORF7a was digested with *Eco*R I and *Xho *I and the DNA fragment containing ORF 7a was cloned into *Eco*R I and *Xho *I sites of pEGFP-C2 (Clontech, Mountain View, USA) resulting in pEGFP-C2-ORF7a where ORF 7a was fused to the C-terminus of EGFP.

The plasmid containing the human full-length Ap_4_A-hydrolase gene was recovered from the yeast clone as described below. The gene was amplified by PCR using the specific primers 5'-GCGGATCCAGATGGCCTTGAGAGCATG-3' and 5'-CGCTCGAGGGGCCTCTATGGAGCAAAGA-3' and subcloned into *Bam*H I and *Xho *I site (underlined) of pcDNA3.1/V5-HisA vector (Invitrogen) to construct pcDNA3.1/Ap_4_A V5-His where V5-His tag was attached to the C-terminus of Ap_4_A-hydrolase. Also, the gene encoding Ap_4_A-hydrolase was subcloned into the *Hind *III and *Eco*R I sites of pDsRed2 (Clontech) (primers 5'-TTAAGCTTATGGCCTTGAGAGCATGTG-3' and 5'-GTGAATTCGTGCCTCTATGGAGCAAAG-3') resulting in pDsRed2-Ap_4_A where DsRed2 was fused to the C-terminus of Ap_4_A-hydrolase. The sequences of all constructs were confirmed by sequencing.

### Yeast two-hybrid screening

The Hybrid Hunter™ yeast two-hybrid kit was purchased from Invitrogen, and all experiments were carried out as recommended by the manufacturer. Briefly, the full-length cDNA of ORF 7a was cloned into pHybLex/Zeo to create a "bait" gene construct pHybLex/Zeo-ORF7a as described above. Hybrid Hunter™ Premade cDNA library (human adult lung) constructed in pYESTrp2 (Invitrogen) was used as a source of "prey" genes.

The plasmid pHybLex/Zeo-ORF7a was used to transform the yeast strain EGY48/pSH18-34, using the lithium acetate method to create a "bait" yeast strain. Next, the "bait" yeast strain was transformed with the cDNA library. Positive clones were initially selected for growth in the absence of uracil, leucine, histidine and tryptophane and further tested for β-galactosidase activity using a filter assay, as suggested by Invitrogen.

Plasmid DNA was isolated from the positive yeast clones. The gene inserts were PCR amplified and then sequenced. Plasmid DNA from positively interacting clones was also re-transformed into the *E. coli *DH5α strain.

### Cell culture and transfection

HEK 293 cells (ATCC CRL-1573) were maintained in minimal Eagle medium (Invitrogen) supplemented with 10% fetal bovine serum, 1% nonessential amino acids, 1% HEPES and 0.05 mg/ml gentamicin (Invitrogen). Cells were cultured at 37° in an incubator supplied with 5% CO_2_.

Cells were transiently transfected with expression vectors using the Profection Mammalian Transfection System-Calcium Phosphate (Promega, Madison, USA), in accordance with the manufacturer's instruction. From 24 to 48 h after transfection, the expression of proteins was screened by Western-blot and confocal microscopic analysis.

### Antibodies

The following antibodies were used in the present study: monoclonal mouse Anti-V5 antibody (Invitrogen), dilution 1:1000; monoclonal mouse anti-HA Tag antibody (Upstate, Temecula, USA), dilution 1:5000; monoclonal mouse anti-β-actin (Sigma-Aldrich, Oakville, Canada), dilution 1:2000; and goat anti-mouse horseradish peroxidase conjugated IgG (BioRad, Mississauga, Canada), dilution 1:2000.

### SDS-PAGE and Western blotting

At 48 h after transfection, HEK293 cells were lysed in RIPA buffer (50 mM Tris-HCl, pH 7.5, 150 mM NaCl, 1% NP-40, 1% deoxicholate Na, 0.1% SDS and 1 mM PMSF). After incubation on ice for 30 min, the samples were briefly sonicated on ice and centrifuged. Supernatants were subjected to 12% SDS-PAGE and were transferred to a nitrocellulose membrane (Ready Gel™ Blotting Sandwiches from BioRad). After incubation with blocking buffer (5% non-fat dry milk in TBST (20 mM Tris-HCl, pH 7.0, 150 mM NaCl, 0.05% Tween-20)), membranes were incubated with specific primary antibodies, washed three times in TBS-T, and then incubated with horseradish-peroxidase-conjugated secondary antibodies. The resulting signals were visualized by the ECL- Plus Western Blotting Detection System (GE Healthcare, Baie d'Urfe, Canada).

### Co- Immunoprecipitation

Cells in 6-well culture plates (5 × 10^5 ^cells per well) were transiently co-transfected with 3 μg pXJ3'-ORF7a-HA and 3 μg pcDNA3.1/V5-His-Ap_4_A using the Profection Mammalian Transfection System-Calcium Phosphate (Promega, Madison, USA). At 48 h after transfection, cells were harvested and washed twice with ice-cold PBS. Cell lysates were prepared in IP-lysis buffer (10 mM HEPES, pH 7.2, 150 mM NaCl, 1 mM PMSF, 1% Nonident-40). The supernatants were incubated with monoclonal Anti-V5 antibody, or anti-HA Tag antibody or anti-β-actin antibody overnight at 4°C. After the incubation, protein A/G Sepharose Fast Flow beads (GE HealthCare) were added and the samples were incubated for additional 1 h at 4°C. The beads were washed five times with IP-lysis buffer, boiled in SDS sample buffer, and subjected to Western blot analysis.

### Confocal microscopy

HEK 293 cells, co-transfected with pDsRed2-Ap_4_A and pEGFP-C2-ORF7a, were grown on 35 mm glass bottom culture dishes (Mattek, Ashland, USA). At 24 h post transfection, cells were analyzed using a confocal microscope Zeiss LSM410 equipped with external Argon Ion Laser. EGFP was excited by Argon Ion Laser Beam (488 nm) while DsRed2 was excited by Helium/Neon Laser beam (594 nm). Both signals were detected simultaneously, and separate images were taken and superimposed.

## List of abbreviations

SARS-CoV: severe acute respiratory syndrome coronavirus; Ap_4_A hydrolase: asymmetrical diadenosine tetraphosphate hydrolase (EC 3.6.1.17); ORF: open reading frame; EGFP: enhanced green fluorescent protein; DsRed: *Discosoma sp*. red fluorescent protein; HEK: human embryo kidney; PBS: phosphate buffered saline; MAPK: mitogen-activated protein kinase; Rb: retinoblastoma; CDK2: cyclin dependent kinase 2.

## Competing interests

The authors declare that they have no competing interests.

## Authors' contributions

AZ conceived of the study and discussed the results. NV and IM performed the experiments, and NV prepared the manuscript.

All authors have read and approved the final manuscript.
